# Effect of Resistance Training Under Normobaric Hypoxia on Physical Performance, Hematological Parameters, and Body Composition in Young and Older People

**DOI:** 10.3389/fphys.2020.00335

**Published:** 2020-04-28

**Authors:** Alexander Törpel, Beate Peter, Lutz Schega

**Affiliations:** Department Health and Physical Activity, Institute III Sport Science, Otto von Guericke University Magdeburg, Magdeburg, Germany

**Keywords:** strength training, strength performance, endurance performance, hypoxic training, intermittent hypoxic resistance training

## Abstract

**Background:**

Resistance training (RT) under hypoxic conditions has been used to increase muscular performance under normoxic conditions in young people. However, the effects of RT and thus of RT under hypoxia (RTH) could also be valuable for parameters of physical capacity and body composition across the lifespan. Therefore, we compared the effects of low- to moderate-load RTH with matched designed RT on muscular strength capacity, cardiopulmonary capacity, hematological adaptation, and body composition in young and older people.

**Methods:**

In a pre–post randomized, blinded, and controlled experiment, 42 young (18 to 30 year) and 42 older (60 to 75 year) participants were randomly assigned to RTH or RT (RTH young, RT young, RTH old, RT old). Both groups performed eight resistance exercises (25–40% of 1RM, 3 × 15 repetitions) four times a week over 5 weeks. The intensity of hypoxic air for the RTH was administered individually in regards to the oxygen saturation of the blood (SpO_2_): ∼80–85%. Changes and differences in maximal isokinetic strength, cardiopulmonary capacity, total hemoglobin mass (tHb), blood volume (BV), fat free mass (FFM), and fat mass (FM) were determined pre–post, and the acute reaction of erythropoietin (EPO) was tested during the intervention.

**Results:**

In all parameters, no significant pre–post differences in mean changes (time × group effects *p* = 0.120 to 1.000) were found between RTH and RT within the age groups. However, within the four groups, isolated significant improvements (*p* < 0.050) of the single groups were observed regarding the muscular strength of the legs and the cardiopulmonary capacity.

**Discussion:**

Although the hypoxic dose and the exercise variables of the resistance training in this study were based on the current recommendations of RTH, the RTH design used had no superior effect on the tested parameters in young and older people in comparison to the matched designed RT under normoxia after a 5-week intervention period. Based on previous RTH-studies as well as the knowledge about RT in general, it can be assumed that the expected higher effects of RTH can may be achieved by changing exercise variables (e.g., longer intervention period, higher loads).

## Introduction

Resistance training is usually used to increase or maintain muscular strength and muscle mass among all age groups. Since age-related loss of muscular strength and muscle mass are associated with an increased manifestation of a lower functional capability (e.g., strength performance, activities of daily living: ADL; Bahat et al., 2018; Wilkinson et al., 2018; Yoshimura et al., 2018), incidence of non-communicable disease (e.g., cardiovascular disease, peripheral artery disease, metabolic disease; Beaudart et al., 2014; Kalyani et al., 2014; Mcleod et al., 2019), and/or mortality (Landi et al., 2013; Volaklis et al., 2015), resistance training is a valuable exercise intervention strategy to conserve or enhance physical performance and health due to the anabolic effects on muscular strength and muscle mass (Kraschnewski et al., 2016; Mcleod et al., 2019).

There are various recommendations for designing exercise variables of resistance training for different age groups (Ratamess et al., 2009; Steib et al., 2010; Garber et al., 2011; Borde et al., 2015; Csapo and Alegre, 2016) which can be used in order to train efficiently and with an adequate stimulus. Moreover, to increase the effectiveness or to achieve additional adaptation effects of resistance training, environmental conditions can also be manipulated. Here, resistance training under hypoxic conditions (RTH; also known as intermittent hypoxic resistance training: IHRT) is a promising type of resistance training attracting increasing attention as an exercise intervention strategy in the last two decades (see meta-analysis by Ramos-Campo et al., 2018b). It is assumed that muscular hypertrophy and muscle strength can be increased to a higher extent with RTH than with traditional resistance training (Feriche et al., 2017). This assumption is reasoned by higher metabolic stress (Scott et al., 2014, 2015a, 2017) triggering functional and structural muscular adaptations (Schoenfeld, 2010, 2013). Even though the exact physiological adaptation processes for RTH are not yet precisely known and understood, it is currently known from 18 international publications on RTH-interventions that this special type of resistance training appears to be partially more effective than resistance training under normoxia regarding the improvement of (i) muscle physiological parameter, (ii) neuromuscular adaptation, (iii) hormonal response, (iv) blood parameter, (v) body composition/body mass, (vi) sprint ability, and (vii) endurance performance/cardiovascular health and fitness (Friedmann et al., 2003; Nishimura et al., 2010; Álvarez-Herms et al., 2012; Manimmanakorn et al., 2013a, b; Álvarez-Herms et al., 2014, 2016; Ho et al., 2014b; Kon et al., 2014; Kurobe et al., 2015; Chycki et al., 2016; Inness et al., 2016; Yan et al., 2016; de Smet et al., 2017; Mayo et al., 2018; Morales-Artacho et al., 2018; Ramos-Campo et al., 2018a; Martínez-Guardado et al., 2019).

So far, RTH has only been studied in young people (age 20 to 30 years), which is why there is no evidence regarding its effects in older people. However, RTH could also be a valuable exercise intervention strategy with a higher effectiveness than a resistance training under normoxic conditions for older people. This assumption is based on study results and the associated discussion that hypoxia and hypoxia in combination with physical exercise (e.g., endurance training, whole body vibration) is a positive evaluated, auspicious intervention strategy against age-related changes of the physical and mental health (Schega et al., 2013, 2016; Bayer et al., 2017; Camacho-Cardenosa et al., 2019a, b) as well as against diseases (e.g., cardio-vascular diseases, metabolic diseases) (Navarrete-Opazo and Mitchell, 2014; Verges et al., 2015; Millet et al., 2016; Serebrovska et al., 2016; Lizamore and Hamlin, 2017; Mallet et al., 2018). Therefore, RTH might be effective to maintain or to increase physical performance and health for older people in order to ensure independent living into old age (Frontera and Bigard, 2002; Hunter et al., 2004; Brady and Straight, 2014). This refers to the effects on the muscular system, cardiopulmonary system, hematological parameters, and body composition.

Therefore, the aim of this study was to investigate the effect of RTH in comparison to matched designed resistance training under normoxia on muscular strength capacity, cardiopulmonary capacity, hematological parameters [erythropoietin (EPO), total hemoglobin mass (tHb), blood volume (BV)], and body composition [fat mass (FM) and fat free mass (FFM)] in young and older people.

Regarding this aim, the design of RTH is decisive. With respect to the current recommendations for RTH, low to moderate loads [∼20 to 50% of the one-repetition maximum (1RM)], medium to high volume (≥3 sets, ≥10 repetitions), and short inter-set pauses (∼30 s) are frequently recommended in order to reach a high level of metabolic stress and therefore to achieve a high stimuli for adaptations of the muscles (Scott et al., 2014, 2015a, 2017). In addition, the number and type of resistance exercises should also be considered. While in previous RTH studies only one or a low number of exercise(s) have frequently been performed, it is more common to include several exercises (singe- and multi-joint) in resistance training due to the usual aim of RT to train muscles or muscle groups of the whole body. With this in mind, we applied a low- to moderate-load RTH with a high volume and a short inter-set pause with several resistance exercises for the muscles of the whole body in comparison to a matched designed RT under normoxia to prove the above-mentioned aim of this study.

## Materials and Methods

### Subjects

A total of 42 young people (18 to 35 years) and 42 older people (60 to 75 years) were recruited using print advertisement in a regional newspaper and with flyers in regional sport and health facilities. To participate in this study, the participants had to fulfill the following requirements: non-smoker; not pregnant; no disorders of the musculoskeletal system, the cardio-pulmonary system, the kidneys, and the brain; no blood donation or loss of blood (>200 ml) in the last 3 months; no sojourn in altitude (>1800 m) in the last 3 months; no additional physical activities other than those during the period of the study. Moreover, the participants were instructed to maintain their regular dietary consumption, to avoid intense exercises, and not to start any nutritional supplementation during the entire study period.

### Study Design

**FIGURE 1:**
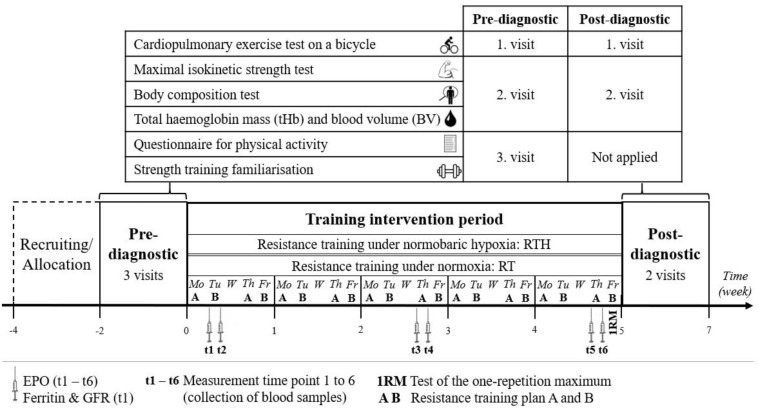
Study design of a 5-week resistance training under hypoxia (RTH) vs. normoxia (RT) with a 2-week pre- and post-diagnostic phase [erythropoietin (EPO) and glomerular filtration rate (GFR)].

**FIGURE 2:**
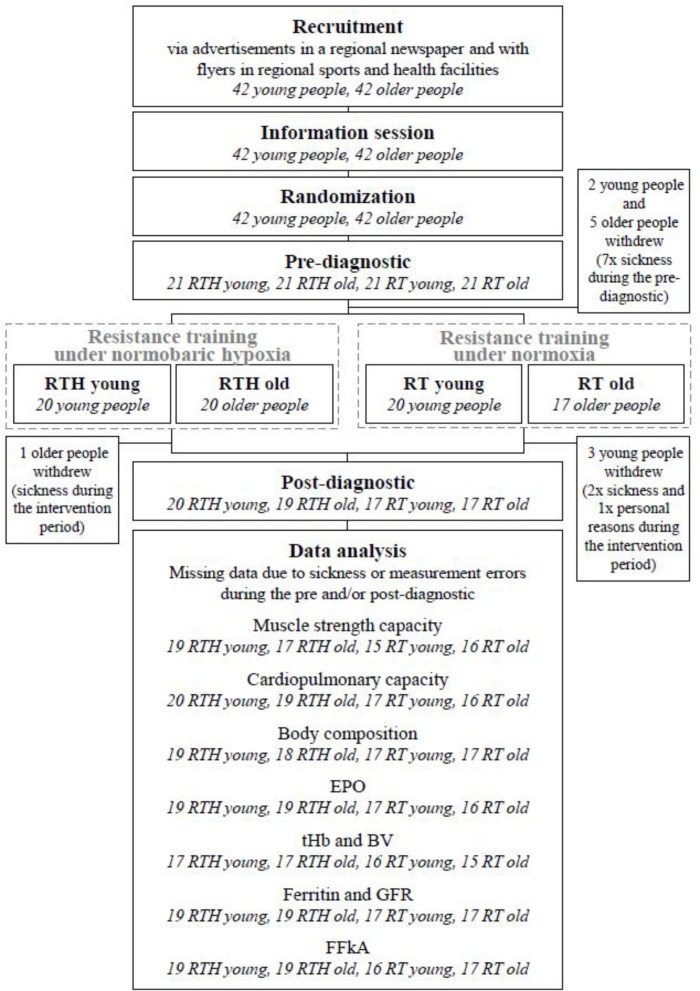
CONSORT flow chart [blood volume (BV), erythropoietin (EPO), glomerular filtration rate (GFR), questionnaire for physical activity “Freiburger Fragebogen zur körperlichen Aktivität” (FFkA), total hemoglobin mass (tHb)].

In this pre-post randomized, blinded, and controlled experiment, the study design described below was carried out a total of four times consecutively (four blocks), twice each with young and older people to include 42 young and 42 older subjects in this study. This was necessary because the planned intervention was limited to the amount of altitude generators and strength training equipment available. Each block started with a recruitment period of 4 weeks. At the end of this period, the participants were invited to visit an information session where the aim and experimental procedure were explained and written informed consent was obtained from each subject. Subsequently, a 2-week pre-diagnostic period and then a 5-week intervention period were conducted. Prior to the start of the pre-diagnostic period, the young and older participants were randomly (permuted block randomization, proportion 1:1, block size = 4; used software: RITA - Randomization In Treatment Arms, Evidat^®^, Germany) assigned to one of the following two groups: resistance training under normobaric hypoxia (RTH young, RTH old) or resistance training under normoxic conditions (RT young, RT old). The intervention period was followed by a 2-week post-diagnostic period (see Figures 1, 2).

The participants were blinded to the hypoxic or normoxic condition (description, see section “Training”) and the staff conducting the diagnostics and analyzing the data was also blinded (double blinded study). The study was officially approved and authorized by the Ethical Committee of the Otto von Guericke University Magdeburg, Germany (Nr. 74/15).

### Training

During the 5-week intervention period, the RTH and RT were conducted four times per week (Monday, Tuesday, Thursday, Friday) within one of three encounter groups each comprised of seven participants (1st group 7.45 am to 10.45 am, 2nd group 11.00 am to 2.00 pm, 3rd group 3.30 pm to 6.30 pm). Prior the start of the study, the participants were able to choose one of these encounter groups in which they were asked to train continuously afterward. Thus, the participants of the RTH and RT were mixed in the three encounter groups. Since the hypoxic air (for the RTH) as well as the normoxic air (for the RT) were individually applied via a facemask (CPAP double-port mask, hand-held or fixed with a bandeau) through an altitude generator (Everest Summit II, Hypoxico^®^, United States), the participants did not know their group affiliation (blinded conditions). Each intervention session had a total duration of 3 h and consisted of three phases lasting 60 min each.

During phases 1 and 3, the participants breathed hypoxic air [phase of intermittent hypoxic exposure (IHE) for the RTH groups] or normoxic air (rest phase for the RT groups) according to their group affiliation in a seated position. With regard to the recommendation by Törpel et al. (2019), the fraction of inspired oxygen (FiO_2_) was individually adjusted to the oxygen saturation of the blood (SpO_2_) for the RTH groups. For the acclimatization to the hypoxic conditions in the RTH groups, the SpO_2_ was gradually reduced: first week 85 to 88%, second to fifth week 80 to 83%. The SpO_2_ was checked every 5–10 min with a pulse oximeter (PO-300, Co. Pulox, China) and the FiO_2_ was adjusted if the SpO_2_ did not meet the predefined SpO_2_-values (after adjustment of the FiO_2_, the SpO_2_ was checked again after 2 min). The RT groups received air with a FiO_2_ of 20.9%. To ensure the blinded conditions, the SpO_2_ was measured in the RT groups like in the RTH groups and sham adjustments were undertaken with the altitude generators. Phase 2 was comprised of supervised resistance training (RTH/RT) with whole-body resistance exercises on resistance training equipment by Proxomed (series compass^®^ 530 from 2011, Germany). Before the resistance training started, a supervised warm-up program of 5 min (dynamic stretching for the major muscle groups) was performed by the participants. To acclimatize to the hypoxic condition, the FiO_2_ was gradually reduced and individually adjusted to a SpO_2_ between 85 and 90% in the first week and to a SpO_2_ between 80 and 85% from the second to the fifth week for the RTH groups. The RT groups continued to receive air with a FiO_2_ of 20.9%. The SpO_2_ was checked in both groups after each exercise and adjusted like in phases 1 and 3.

The resistance training program consisted of two training plans (A and B) that were performed alternately. Training plans A and B each included multiple-set circuit training with eight machine-based resistance exercises: Training plan A: (i) lat pulldown, (ii) butterfly reverse, (iii) upper body extension, (iv) knee flexion, (v) vertical row, (vi) upper body rotation, (vii) biceps curl left, (viii) biceps curl right; Training plan B: (i) triceps press, (ii) leg press, (iii) butter fly, (iv) upper body flexion, (v) knee extension, (vi) chest press, (vii) shoulder side rises left, and (viii) shoulder side rises right. The order of performing the exercises was the same for each participant, but due to the fact that all participants trained at the same time, each one had a different start-exercise and therefore an individual sequence. Each exercise of the training plans was performed with three sets of 15 repetitions, a cadence of 2-0-2-1 s (a metronome with 60 beats per minute was used), an inter-set pause of 30 s, with full range of motion, and with a load that was individually adjusted to a subjective perceived exertion using the CR-10 scale (value 7 [very hard]; Morishita et al., 2013, 2018, 2019). The participants were asked about their subjective perceived exertion at the end of the third set of each resistance exercise and in each training session. Since the load was set to a submaximal value of subjective perceived exertion, resistance training was not performed to muscular failure. The inter-exercise pause lasted 2 min. The used resistance exercise variables were designed to reach a high amount of metabolic stress (see introduction) in order to increase the adaptational effects of resistance training (de Freitas et al., 2017; Feriche et al., 2017). Here, we took the recommendations by Scott et al. (2015a) into account.

### Pre-Post-Diagnostic and Resistance Training Familiarization

Within the pre-diagnostic phase, each participant had three visits to the lab separated by at least 3 days. At the first visit, the participant performed a cardiopulmonary exercise test (CPET) on a bicycle ergometer. An isokinetic maximal strength test and a body composition test as well as the determination of total hemoglobin mass (tHb) and blood volume (BV) were performed at the second visit. The participants completed a questionnaire on physical activity and got an introduction to the training plan at their third visit (see Figure 1).

#### Cardiopulmonary Exercise Test

The cardiopulmonary capacity was tested with an incremental load test (initial load 50 W, 25 W increment at every 3 min, cadence 70 to 80 min^–1^) on a bicycle ergometer (Xrcise Cycle Med, Cardiowise^®^, Germany) in combination with spirometry (MetaMax 3B R2, Co. CORTEX Biophysik, Germany). The test was performed until subjective maximal physical exhaustion. During the whole test, vital parameters (e.g., heart rate, blood pressure) and heart rhythm (measured by a 12-channel ECG, AT104 PC, Co. SCHILLER Reomed AG, Switzerland) were observed by a medical doctor. Blood pressure was measured (manual auscultatory measurement, in regard of Currie et al., 2018) at rest and at the last 30 s in each stage. The following outcomes of the CPET were analyzed: maximal oxygen consumption (VO_2__peak_), oxygen consumption at 1 watt/kg body mass (VO2_1__W/kg_), maximal power output (PO_max_), power output at the second lactate threshold (PO_*LT*__2_) (the second lactate threshold was determined with the D-max method; Czuba et al., 2009; Jamnick et al., 2018), heart rate at 1 watt/kg body mass (HR_1__W/kg_), and the systolic and diastolic blood pressure (SBP, DBP) at rest and at these watt-stages which were performed by all participants.

#### Isokinetic Maximal Strength Test

The isokinetic maximal strength (F_max_) was tested for the exercises elbow flexion and elbow extension as well as knee extension and knee flexion for the dominant and non-dominant extremities using the BTE PrimusRS (Co. Baltimore Therapeutic Equipment Company, United States). The test procedure and the data processing were conducted following the recommendations by Törpel et al. (2017). The F_max_ was first tested for the lower extremities and then for the upper extremities. The test procedure started with a 5-min warm-up for the lower extremities on a bicycle ergometer (KardiomedBike, Co. Proxomed^®^, Germany) and for the upper extremities on an arm crank ergometer (Lode Angio, Co. Lode BV Technologies, Netherlands) with a load of 1 W/kg and 0.5 W/kg (each with a cadence of ∼70 min^–1^). The test procedure was carried out as follows: warm-up for the lower extremities, F_max_ test dominant side for the exercise knee extension and flexion, 3 min rest, F_max_ test non-dominant side for the exercise knee extension and flexion, warm-up for the upper extremities, F_max_ test dominant side for the exercise elbow extension and flexion, 3 min rest, F_max_ test non-dominant side for the exercise elbow extension and flexion. The F_max_ tests were performed with two familiarization sets of five repetitions (1st set passive, 2nd set with estimated 50% of the 1RM, pause in between: 30 s after the 1st set and 90 s after the 2nd set). Thereafter, the F_max_ tests were performed with five repetitions. The movement velocity was 60°/s in each set (the used test variables are established for this kind of diagnostic, see Pereira and Gomes, 2003; Pincivero et al., 2003). To further analyze the data, the values of the dominant side and non-dominant side were averaged. The F_max_ corresponds the mean torque of the three highest out of five repetitions (Müller et al., 2007).

#### Body Composition Test

The body composition [fat mass (FM), fat free mass (FFM)] was determined with the bioelectrical impedance analysis test (BIA; Kyle, 2004) using Nutriguard-MS (Co. Data Input, Germany). The participants were asked to fast for 4 h before the body composition test was performed. The body composition test started at least 20 min after the isokinetic maximal strength test. After bladder voiding and a period of 10 min in a supine position, the electrical resistance between the hand and the foot was measured by placing electrodes on the right wrist and back of the hand as well as the right ankle and the back of the foot. To calculate body components with the software NutriPlus (Co. Data Input, Germany), the body weight and height of the participants were determined immediately before the test (used weighing scale with stadiometer: seca 764, Co. seca, Germany) (Khalil et al., 2014).

#### Hematological Parameters

The hematological parameters tHb and BV were determined by the carbon monoxide (CO) rebreathing method by Falz et al. (2009, 2014). The test–retest reproducibility of this method is 1.6% (typical error) (Falz et al., 2009). The administered CO bolus had a dosage of 1 ml/kg FFM for females and 1 ml/kg body mass for males and was rebreathed for 15 min. Capillary blood samples from the earlobe were drawn twice immediately prior to the bolus and at the end of the 1^st^, 9^th^, 11^th^, 13^th^, and 15^th^ minute during the rebreathing period to analyze hemoglobin, hematocrit, and the fraction of carboxyhemoglobin (used blood gas analyzer: ABL80 FLEX CO-OX, Co. Radiometer, Denmark).

#### Physical Activity

The physical activity of the participants prior to the intervention was determined using the German questionnaire “Freiburger Fragebogen zur körperlichen Aktivität” (FFkA). The FFkA determines the health-related physical activity retrospective for 1 week (in hours per week) (Frey et al., 1999). Here, a distinction is made between basic activity (FFkA_basic_), extracurricular activity (FFkA_extracurr_), sports activity (FFkA_sports_), and the sum of the total activity (FFkA_total_). To monitor the physical activity during the intervention period, the participants received an accelerometer (GT3X, Co. Actigraph, United States) which was worn on the left wrist. Due to the maximum recording period of the GT3X of 3 weeks, just the first 3 weeks of the intervention period were monitored. The GT3X calculated the kilocalorie per day (kcal/d) and the metabolic equivalent per day (MET/d). Wearing of the accelerometer was elective for the participants and a total of 10 participants decided not to wear the GT3X (participated subjects: RTH young *n* = 15, RT young *n* = 13, RTH old *n* = 18, RT old *n* = 17). Because it is known that physical performance and adaptation processes are related to physical activity (Warburton et al., 2015), we used the FFkA and the GT3X to characterize the level of physical activity between the RTH group and RT group to ensure that there were no significant differences.

#### Familiarization to the Strength Training

The introduction phase in the strength training plan aimed to familiarize the participants with the training procedure and included: (i) the individual determination of an adequate anthropometric setting of the strength training equipment, (ii) the explanation of the execution of each exercise, and (iii) the initial determination of the load for each resistance exercise for a subjectively perceived effort of 7 on the CR-10 scale. These were conducted by a sport scientist who was licensed in exercise training for public health, prevention, and rehabilitation and attended a course for using the strength training equipment by Proxomed^®^. The pre-diagnostic methods of visit one and two were adequately conducted in the post-diagnostic (see Figure 1).

### Diagnostic During the Training Intervention

#### Erythropoietin and Control Parameters

We collected blood samples from the participants immediately before and after the 2^nd^ (t1, t2), 11^th^ (t3, t4), and 19^th^ (t5, t6) of the 20 training sessions to determine the acute reaction (t1 to t2, t3 to t4, t5 to t6) of the hematopoietic hormone erythropoietin (EPO) (determined in the blood serum). Moreover, ferritin and the glomerular filtration rate (GFR) were determined in t1 to check the iron level and renal function (both important for erythropoiesis; Jin et al., 2008; Wang et al., 2010) (see Figure 1).

A 10 ml venous blood sample was drawn from a superficial forearm vein under stasis conditions by a medical doctor. The blood samples were analyzed in a local medical laboratory.

#### One-Repetition Maximum

To be able to compare the used load as percentage of the 1RM from this study with other RTH-studies, we quantified the 1RM exemplary for the exercises of the legs (knee extension, knee flexion) and arms (biceps curl, triceps press) during the 20^th^ training session immediately before the resistance training (see Figure 1). Here we used the equation by Mayhew et al. (1992) to predict the 1RM by using the number of repetitions until maximal exhaustion with a submaximal load.

### Statistical Analysis

The data was checked for normal distribution using the Kolmogorov-Smirnov-Test. For normally distributed data, the mean and standard deviation and for non-normally distributed data the median and the interquartile range is reported.

Differences between two groups were analyzed by using an unpaired *t*-test or Mann–Whitney *U*-test (group effect). To check the homogeneous distribution of the sex within and across groups, we conducted a chi-squared test. In a primary analysis, the effect of the RTH vs. the RT on the parameters of the cardiopulmonary capacity, the muscular strength capacity, EPO, the hematological parameter tHb and BV, and the body composition (FM, FFM) were analyzed by performing a two-way ANOVA with repeated measures (time × group effect). Since multiple tests can lead to false-positive results (Sainani, 2009), we corrected all *p*-values (Bonferroni–Holm method; Aickin and Gensler, 1996) of the primary analysis (time × group effects) that belong to the same parameter group (e.g., cardiopulmonary capacity). In a secondary analysis, we checked the changes of the parameters within each single group (RTH young, RT young, RTH old, RT old) by using a one-way ANOVA with repeated measures (time effect).

To compare the loads as percentage of the 1RM between the four groups, a one-way ANOVA without repeated measures (main group effect) was performed. When a significant effect was identified, the Bonferroni *post hoc* test was performed to localize the differences between the groups (group effect).

In conjunction with the time × group effects, the effect size partial eta squared ($\eta_{\text{p}}^{2}$) will be reported (Lakens, 2013) and rated as small for 0.1 to 0.3, medium for 0.3 to 0.5, and large for over than 0.5 (Bakeman, 2005). Statistical significance was set at *p* < 0.05 for all analyses. The data processing and analyses were done using the software Excel (Microsoft office 365, Microsoft, United States) and SPSS Statistics (Statistical Package for social science, Version 24, IBM^®^, United States).

## Results

### Participants

A total of 73 out of 84 participants finished the whole study (see Figure 2). These 73 participants had a good attendance-rate to the 20 training sessions (∼94%).

In addition to the participants who dropped out, we had occasional data losses in some of the used methods because some participants became acutely ill during the post-diagnostic period as well as due to some measurement errors in the pre- and/or post-tests (see Figure 2).

The personal data of the participants (age, height, body weight, body mass index [BMI]) were similar between the groups RTH and RT within the age cohorts (Table 1). The same applies to the parameters ferritin and GFR, the physical activity prior (shown by the FFkA) and during (shown by the GT3X) intervention period (Table 1).

**TABLE 1:** Base line data of the participants (data are shown as mean ± standard deviation or median [interquartile range]).

	Young people			Older people		
	RTH	RT	*p*	RTH	RT	*p*
*N* (*n* female, *n* male)	20 (f 3, m 17)	17 (f 2, m 15)	0.774#	19 (f 10, m 9)	17 (f 8, m 9)	0.738#
Age [years]	24.5 ± 4.5	24.0 ± 3.6	0.741*	68.1 ± 4.6	67.8 ± 4.1	0.845*
Height [m]	1.80 ± 0.08	1.78 ± 0.06	0.531*	1.66 ± 0.08	1.67 ± 0.08	0.935*
Body weight [kg]	75.5 ± 7.8	76.3 ± 9.2	0.756*	76.6 ± 14.7	75.2 ± 14.9	0.780*
BMI [kg/m^2^]	23.5 ± 2.6	24.1 ± 2.5	0.479*	27.6 ± 4.2	26.9 ± 3.6	0.637*
Ferritin [ng/ml]	85 [32]	67 [116]	0.962**	90 [138]	150 [237]	0.233**
GFR [ml/min/1,73 m^2^]	103 ± 13	104 ± 14	0.825*	71 ± 14	80 ± 12	0.058*
FFkA_basic_	2.4 [2.3]	2.2 [1.9]	1.000**	7.2 [17.7]	9.3 [14.8]	1.000**
FFkA_extracurr_	1.8 [3.5]	1.0 [2.9]	1.000**	2.2 [4.1]	1.5 [4.3]	0.890**
FFkA_sports_	4.0 [3.2]	7.0 [7.2]	0.118**	1.8 [2.7]	1.0 [2.3]	0.446**
FFkA_total_	8.6 [4.9]	9.9 [10.1]	0.610**	16.4 [22.8]	13.1 [15.3]	0.534**
GT3X kcal/d	1096 ± 310	1165 ± 506	0.673*	1433 ± 441	1402 ± 441	0.836*
GT3X MET/d	1.40 ± 0.12	1.42 ± 0.17	0.711*	1.50 ± 0.12	1.51 ± 0.12	0.907*

[glomerular filtration rate of the kidney (GFR), physical activity in hours per week prior to the intervention: basic activity (FFkA_basic_), extracurricular activity (FFkA_extracurr_, sports activity: FFkA_sports_, total activity: FFkA_total_), and physical activity in kilocalories and metabolic equivalent per day (kcal/d, MET/d) during the first 3 weeks of the intervention period; f: female, m: male]. ^#^Chi-square test to analyze the homogeneous distribution of females and males between the groups RTH and RT within the cohort of young and older people. *t-test; **U-test.

Gender distribution was homogeneous within the age-groups (chi-squared results, see Table 1) but not across all groups (*p* = 0.010).

### Load of the Resistance Exercises

**FIGURE 3:**
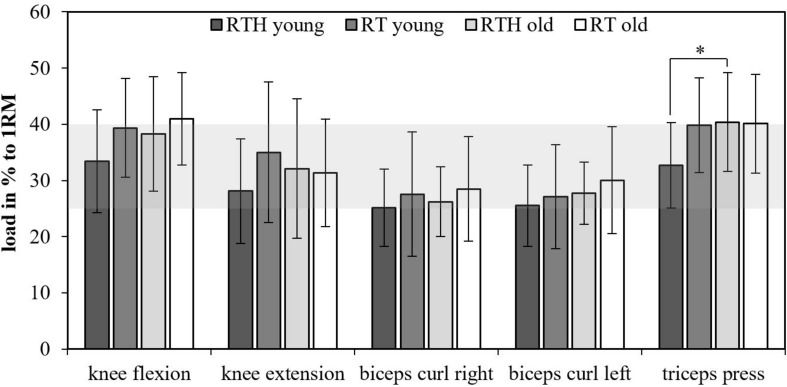
Loads as percentage to the one-repetition maximum (1RM); resistance training under normobaric hypoxia (RTH), resistance training under normal conditions (RT); the gray area marks the mean range of the used loads for the resistance exercises in relationship to the 1RM (group effects: ^∗^*p* ≤ 0.050, ^∗∗^*p* ≤ 0.010, ^∗∗∗^*p* ≤ 0.001).

The values of the used loads in the groups RTH and RT of the young and older participants are shown in Figure 3 as percentage of the 1RM. The used loads for the resistance exercises ranged between 25 and 40% (up to 50% with respect to the standard deviation) of the 1RM. Except for the resistance exercise triceps press (RTH young vs. RTH old: *p* = 0.037), there were no significant differences for the used loads within the resistance exercises between the four groups. The difference of the used loads between the four groups was maximal 8% (RTH young vs. RT old for the exercise knee extension) and usually only between 2 and 5%.

### External and Internal Hypoxic Intensity

**FIGURE 4:**
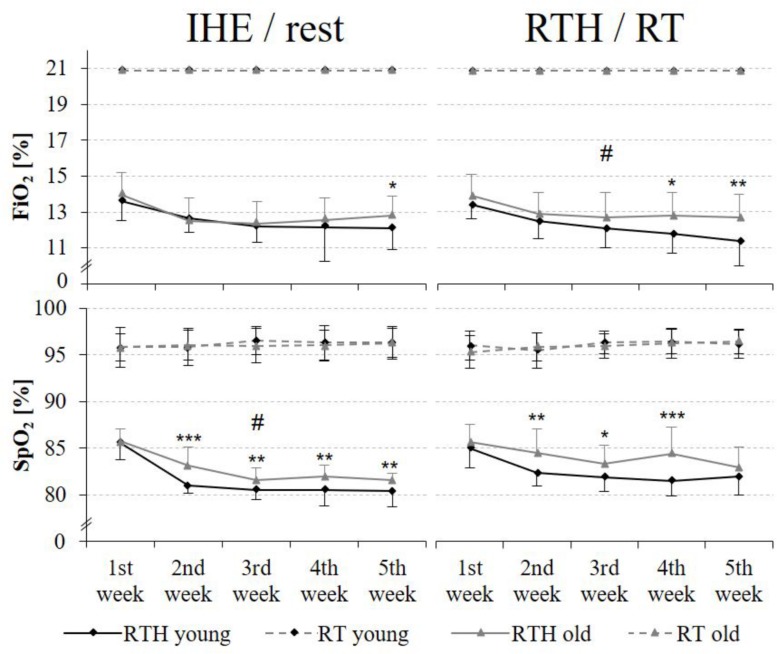
External [fraction of inspired oxygen (FiO_2_)] and internal [oxygen saturation of the blood (SpO_2_)] hypoxic intensity during the IHE/rest phase and the RTH/RT phase in each week of the 5-week intervention period; resistance training under hypoxic conditions (RTH), resistance training under normoxic conditions (RT), intermittent hypoxic exposure (IHE) (time × group effects: #*p* ≤ 0.050, ##*p* ≤ 0.010, ###*p* ≤ 0.001; group effects between the groups RTH young and RTH old: *p ≤ 0.050, **p ≤ 0.010, ***p ≤ 0.001).

The external hypoxic intensity (= FiO_2_) was comparable over the 5-week training intervention period during the IHE phase but significantly lower during the RTH phase for the young age cohort in comparison to the older age cohort of the RTH groups (RTH young vs. RTH old: time × group effect *F* = 3.099, *p* = 0.041, $\eta_{\text{p}}^{2}$ = 0.077, see Figure 4). The internal hypoxic intensity (= SpO_2_) was also lower in the group RTH young than in the group RTH old (see Figure 4), however, these differences were only statistically significant during the IHE phase over the 5 weeks (time × group effect *F* = 3.074, *p* = 0.029, $\eta_{\text{p}}^{2}$ = 0.077). In a differentiated analysis for each week, the FiO_2_ was significantly lower in the group RTH young than for the RTH old at the 5^th^ week during the IHE phase (*p* = 0.047) as well as at the 4^th^ (*p* = 0.020) and 5^th^ (*p* = 0.005) week during the RTH phase. From week 2 to 5 (2^nd^ week: *p* < 0.001; 3^rd^ week: *p* = 0.007; 4^th^ week: *p* = 0.002; 5^th^ week: *p* = 0.010) and during the RTH phase from week two to four (2^nd^ week: *p* = 0.005; 3^rd^ week: *p* = 0.014; 4^th^ week: *p* = 0.001), the SpO_2_ was significantly lower for the group RTH young in comparison to the group RTH old (see Figure 4) during the IHE phase. There was no significant difference regarding the SpO_2_ between the RT groups of the young and older people over the 5-week training intervention period.

### Cardiopulmonary Capacity

The cardiopulmonary exercise tests were performed in the pre- and post-diagnostic until maximal physical exhaustion (respiratory exchange ratio: RER young participants ≥ 1.10; RER older participants ≥ 1.00 to ≥ 1.05; see Edvardsen et al., 2014) by the young and older participants (see Tables 2, 3). We observed significantly higher values of the RER in the post-tests then in the pre-tests for the groups RTH young and RT young (see time effect in Table 2) but these higher values were not strictly associated with a higher cardiopulmonary capacity. The parameters VO2_peak_, VO2_1__W/kg_, PO_max_, PO_*LT*__2_, and HR_1__W/kg_ did not change differently between group RTH and group RT within the age cohorts (see time × group effects in Tables 2, 3). However, regarding the young cohort, the VO2 at 1W/kg was significantly reduced for the group RTH (see time effect in Table 2) and the submaximal HR at 1W/kg was significantly reduced for the group RT. Regarding the older age cohort, we observed a significantly higher VO2_peak_ and PO_max_ as well as a significantly lower HR at 1W/kg in the RTH group after the 5-week intervention period. The increase of the VO2_peak_ (+ 10.5%) in the group RT old only narrowly missed a significant effect (see time effect in Table 3).

**TABLE 2:** Pre–post results of the cardiopulmonary capacity, muscular strength capacity, hematological parameters, and body composition of the group RTH young (resistance training under hypoxia) and RT young (resistance training under normoxia).

Young people	Group	Pre	Post	Pre–post change [%]	Time effect			Time × group effect*		
		Mean ± SD	Mean ± SD		*F*	*p*	$\eta_{\text{p}}^{2}$	*F*	*p*	$\eta_{\text{p}}^{2}$
**Cardiopulmonary capacity**										
RER_max_	RTH	1.09 ± 0.05	1.14 ± 0.05	+4.6	11.074	**0.004**	0.381	0.083	1.000	0.002
	RT	1.05 ± 0.06	1.11 ± 0.05	+5.7	9.529	**0.007**	0.373			
VO2_peak_ [l/min]	RTH	3.14 ± 0.62	3.14 ± 0.46	0.0	0.002	0.969	0.000	0.248	1.000	0.007
	RT	3.34 ± 0.67	3.25 ± 0.61	–2.7	0.830	0.376	0.049			
VO2_1__W/kg_ [l/min]	RTH	1.49 ± 0.26	1.42 ± 0.15	–4.7	6.797	**0.018**	0.274	1.493	1.000	0.042
	RT	1.52 ± 0.16	1.50 ± 0.21	–1.3	0.460	0.507	0.028			
O_max_ [W]	RTH	245 ± 49	250 ± 43	+2.0	3.003	0.100	0.143	0.038	1.000	0.001
	RT	240 ± 49	244 ± 47	+1.6	1.214	0.287	0.171			
PO_LT__2_ [W]	RTH	170 ± 33	173 ± 29	+2.0	1.421	0.250	0.077	0.061	1.000	0.007
	RT	160 ± 31	164 ± 32	+2.8	1.921	0.185	0.107			
HR_1__W/kg_ [min^–1^]	RTH	118 ± 16	112 ± 11	–4.7	2.728	0.118	0.146	0.142	1.000	0.004
	RT	116 ± 16	109 ± 11	–6.6	7.855	**0.013**	0.329			
**Muscular strength capacity**										
F_max_ elbow ext. [Nm]	RTH	43 ± 9	42 ± 10	–2.3	1.235	0.281	0.064	2.631	0.460	0.076
	RT	42 ± 12	45 ± 13	+6.7	1.367	0.262	0.089			
F_max_ elbow flex. [Nm]	RTH	50 ± 13	49 ± 12	–3.4	4.255	0.054	0.191	5.127	0.120	0.138
	RT	48 ± 12	51 ± 14	+6.1	1.960	0.183	0.123			
F_max_ knee ext. [Nm]	RTH	140 ± 18	143 ± 22	+2.0	1.900	0.185	0.095	1.689	0.812	0.050
	RT	142 ± 20	149 ± 13	+5.0	6.776	**0.021**	0.326			
F_max_ knee flex. [Nm]	RTH	104 ± 25	112 ± 29	+7.1	19.803	**0.007**	0.343	3.469	0.288	0.098
	RT	103 ± 24	116 ± 20	+13.0	43.871	**<0.001**	0.758			
**Hema-tology**										
tHb [g]	RTH	1037 ± 180	1011 ± 172	–2.5	5.916	**0.027**	0.270	0.790	0.762	0.025
	RT	1010 ± 193	1000 ± 204	–1.0	0.614	0.446	0.039			
BV [ml]	RTH	7145 ± 1033	6971 ± 1025	–2.4	2.369	0.143	0.129	1.093	0.608	0.034
	RT	6727 ± 1116	6720 ± 1162	–0.1	0.005	0.947	0.000			
**Body comp.**										
FM [kg]	RTH	9.6 ± 4.9	9.4 ± 4.8	–2.1	1.380	0.255	0.071	2.034	0.489	0.056
	RT	9.7 ± 4.2	9.9 ± 4.3	+2.1	0.782	0.390	0.047			
FFM [kg]	RTH	66 ± 8	65 ± 8	–0.6	1.813	0.195	0.091	1.081	0.918	0.031
	RT	67 ± 10	67 ± 10	+0.1	0.060	0.810	0.004			

(*corrected p-value by using the Bonferroni–Holm method – the correction factor corresponds to the number of the tests [time × group effect] within the parameter groups; maximal respiratory exchange ratio: RER_max_, peak oxygen uptake: VO2_peak_, oxygen uptake at one watt per kilogram: VO2_1__W/kg_, maximal power output: PO_max_, power output at the second lactate threshold: PO_LT__2_, heart rate at one watt per kilogram: HR_1__W/kg_, maximal muscular strength: F_max_ for the exercises elbow extension and flexion/knee extension and flexion, total hemoglobin mass: tHb, blood volume: BV, fat mass: FM, fat free mass: FFM). P-values in bold show a significance of p ≤ 0.050.

**TABLE 3:** Pre–post results of the cardiopulmonary capacity, muscular strength capacity, hematological parameters, and body composition of the group RTH old (resistance training under hypoxia) and RT old (resistance training under normoxia).

Older people	Group	Pre	Post	Pre–post change [%]	Time effect			Time × group effect^∗^		
		Mean ± SD	Mean ± SD		*F*	*p*	$\eta_{\text{p}}^{2}$	*F*	*p*	$\eta_{\text{p}}^{2}$
**Cardiopulmonary capacity**										
RER_max_	RTH	1.11 ± 0.06	1.11 ± 0.07	0.0	0.077	0.785	0.004	0.046	1.000	0.001
	RT	1.12 ± 0.08	1.11 ± 0.09	–0.9	0.394	0.540	0.026			
VO2_peak_ [l/min]	RTH	2.02 ± 0.53	2.17 ± 0.71	+7.4	4.499	**0.048**	0.200	0.257	1.000	0.008
	RT	1.91 ± 0.64	2.11 ± 0.66	+10.5	4.492	0.051	0.230			
VO2_1__W/kg_ [l/min]	RTH	1.39 ± 0.26	1.42 ± 0.28	+2.2	0.750	0.399	0.042	1.243	1.000	0.040
	RT	1.40 ± 0.31	1.51 ± 0.45	+7.9	2.945	0.110	0.185			
PO_max_ [W]	RTH	138 ± 44	145 ± 44	+5.2	7.299	**0.015**	0.289	1.456	1.000	0.042
	RT	122 ± 43	125 ± 41	+2.2	1.130	0.305	0.070			
PO_LT__2_ [W]	RTH	104 ± 28	108 ± 27	+3.8	3.692	0.074	0.198	0.196	1.000	0.227
	RT	94 ± 21	100 ± 25	+5.9	4.041	0.067	0.252			
HR_1__W/kg_ [min^–1^]	RTH	118 ± 20	116 ± 21	–1.9	6.755	**0.019**	0.284	0.564	1.000	0.019
	RT	117 ± 14	117 ± 14	0.0	1.394	0.261	0.104			
**Muscular strength capacity**										
F_max_ elbow ext. [Nm]	RTH	30 ± 10	31 ± 12	+3.7	1.715	0.209	0.097	1.093	1.000	0.034
	RT	31 ± 10	31 ± 10	–0.3	0.005	0.946	0.000			
F_max_ elbow flex. [Nm]	RTH	33 ± 10	34 ± 12	+4.6	3.700	0.072	0.188	0.090	1.000	0.003
	RT	32 ± 10	33 ± 11	+3.7	3.094	0.099	0.171			
F_max_ knee ext. [Nm]	RTH	98 ± 20	102 ± 22	+4.8	3.139	0.095	0.164	2.560	0.480	0.079
	RT	95 ± 23	105 ± 23	+10.5	26.851	**<0.001**	0.657			
F_max_ knee flex. [Nm]	RTH	59 ± 15	66 ± 20	+11.9	10.175	**0.006**	0.389	0.312	1.000	0.010
	RT	62 ± 18	71 ± 21	+14.1	19.803	**<0.001**	0.569			
**Hema-tology**										
tHb [g]	RTH	818 ± 152	815 ± 153	–0.4	0.128	0.725	0.008	0.078	1.000	0.003
	RT	842 ± 248	835 ± 235	–0.8	0.372	0.552	0.026			
BV [ml]	RTH	5938 ± 1064	5928 ± 1040	–0.2	0.016	0.902	0.001	0.005	1.000	0.000
	RT	5916 ± 1387	5914 ± 1345	0.0	0.000	0.986	0.000			
**Body comp.**										
FM [kg]	RTH	18 ± 9	18 ± 9	–0.6	0.082	0.778	0.005	0.076	1.000	0.002
	RT	17 ± 7	17 ± 7	–0.6	0.024	0.878	0.002			
FFM [kg]	RTH	59 ± 12	60 ± 13	+0.3	0.525	0.479	0.030	0.344	1.000	0.010
	RT	58 ± 13	58 ± 13	0.0	0.007	0.936	0.000			

(*corrected p-value by using the Bonferroni–Holm method – the correction factor corresponds to the number of the tests [time × group effect] within the parameter groups; maximal respiratory exchange ratio: RER_max_, peak oxygen uptake: VO2_peak_, oxygen uptake at one watt per kilogram: VO2_1__W/kg_, maximal power output: PO_max_, power output at the second lactate threshold: PO_LT__2_, heart rate at one watt per kilogram: HR_1__W/kg_, maximal muscular strength: F_max_ for the exercises elbow extension and flexion/knee extension and flexion, total hemoglobin mass: tHb, blood volume: BV, fat mass: FM, fat free mass: FFM). P-values in bold show a significance of p ≤ 0.050.

**FIGURE 5:**
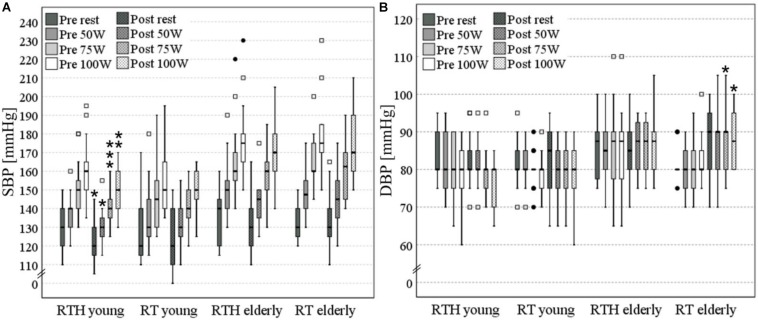
Systolic **(A)** and diastolic **(B)** blood pressure (SBP, DBP) at rest, 50, 75, and 100 W [significant pre-post- differences are marked with an asterisk (*) above the box plots: one *p ≤ 0.050, two vertical *p ≤ 0.010, three vertical *p ≤ 0.001; values with a distance of the 1^st^ or 3^rd^ quartile of more than 1.5-fold (□) or 3-fold (•) of the interquartile range are separately shown as outliner].

Due to the fact that all participants performed the CPET to at least 100 W, we analyzed the SBP and DBP at rest, 50, 75, and 100 W. After the 5-week intervention, the group RTH young had significantly lower values of the SBP at rest (−10 mmHg: *p* = 0.011) and 50 W (−10 mmHg: *p* = 0.012), 75 W (−10 mmHg: *p* = 0.001), and 100 W (−10 mmHg: *p* = 0.008) during the cardiopulmonary exercise test. The DBP was significantly higher from pre to post in the group RT old at 75 W (+ 10 mmHg: *p* = 0.030) and 100 W (+ 7.5 mmHg: *p* = 0.039) (see Figure 5).

### Muscular Strength Capacity

We found no significant differences regarding the changes of the maximal isokinetic muscular strength between the groups RTH and RT within the young or older age cohorts (see time × group effects in Tables 2, 3). However, the intervention led to a significant improvement in muscular strength of the lower extremities (5 to 14%) for the groups RT young (knee extension), RTH old (knee extension, knee flexion), and RT old (knee extension, knee flexion) (see time effects in Tables 2, 3).

### EPO and Hematological Parameters

**FIGURE 6:**
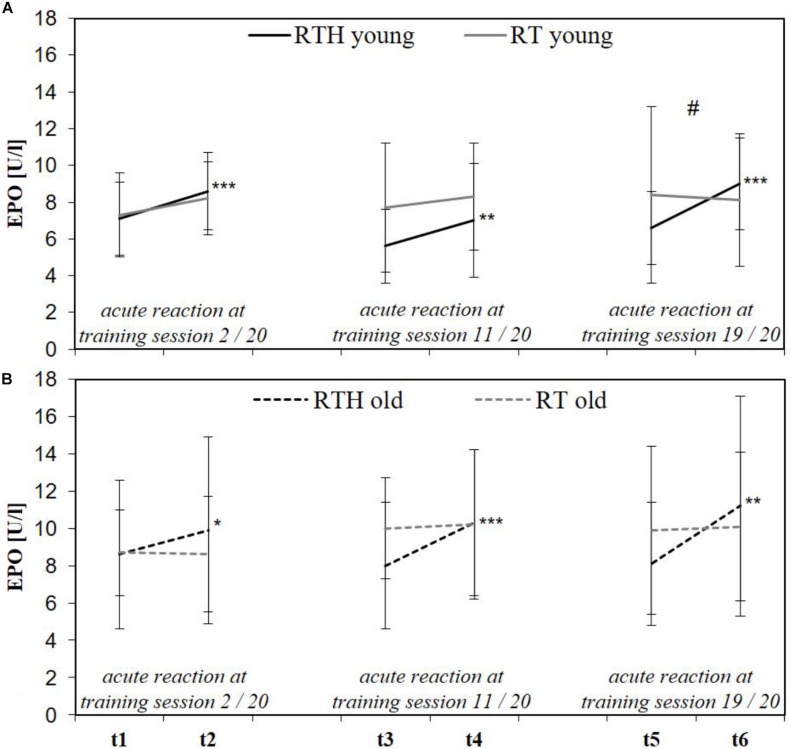
Acute reaction of the erythropoietin (EPO) immediately before and after the 2nd (t1, t2), 11th (t3, t4), and 19th (t5, t6) of 20 training sessions in the young **(A)** and older **(B)** age cohort of the group RTH and RT [resistance training under normobaric hypoxia (RTH), resistance training under normoxia (RT); time × group effect: #*p* ≤ 0.050, ##*p* ≤ 0.010, ###*p* ≤ 0.001; time effect: *p ≤ 0.050, **p ≤ 0.010, ***p ≤ 0.001].

Both RTH young and RTH old increased EPO significantly at the beginning, the middle, and the end of the intervention period (see time effects in Figure 6). However, a significant difference to the acute response of EPO was only identified at the end of the intervention and only in the young participants (time × group effect: *F* = 12.697, *p* = 0.003, $\eta_{\text{p}}^{2}$ = 0.278; see Figure 6).

The small changes from pre to post regarding tHb and the BV were not significantly different between the groups RTH and RT within the age cohorts (see time × group effects in Tables 2, 3). However, a significantly lower tHb value was observed for the group RTH young (see time effect in Tables 2, 3).

### Body Composition

The body composition (FM, FFM) was not affected by the 5-week RTH or RT for the young or older subjects (see Tables 2, 3).

## Discussion

Regarding the primary analysis, we found no superior effects of RTH in comparison to matched designed RT on physical performance (cardiopulmonary and muscle strength capacity), tHb, BV, and body composition (FFM, FM). Heterogeneous results regarding the effect of this type of RTH are currently available: higher effectiveness of the RTH regarding the improvement of muscle hypertrophy (Nishimura et al., 2010), muscle strength capacity (de Smet et al., 2017; Martínez-Guardado et al., 2019), and anaerobic performance (Álvarez-Herms et al., 2014) vs. no superior effects regarding muscle hypertrophy, muscle strength endurance capacity, maximal muscular strength, muscle fiber type distribution, the vascular endothelial growth factor (VEGF), and various enzymes and muscle specific proteins (Friedmann et al., 2003; Ho et al., 2014b). These incongruent results might be reasoned by the differences regarding the designs of the resistance training as well as the hypoxic doses of these studies (including the current study). Moreover, the researched effects on the mentioned parameters have been demonstrated only once. Therefore, further studies are needed that confirm (reproduce) these results. Based on this, the results of the current study and the previous studies are not necessarily generalizable for RTH (with low to moderate loads). Due to the low number of RTH intervention studies using low to moderate loads, an adequate dose–response relationship (resistance training and hypoxic dose) which would be required to apply an effective stimulus to achieve a superior effect to an equal designed resistance training under normoxia for young and older people cannot be identified at present. Since the stimuli of both resistance training (Toigo and Boutellier, 2006) and hypoxia (Azad et al., 2017) are quite complex (thus probably also their interaction) and the extent of adaptational processes caused by physical exercise (e.g., resistance training;Erskine et al., 2010; Ahtiainen et al., 2016; Ross et al., 2019) or hypoxic training (Chapman, 2013) are interindividual variable (responder vs. non-responder; Gabriel and Zierath, 2017), it is hard to design a RTH that is both generally effective as well as superior in comparison to a resistance training under normoxia. We tried to do this by considering the current recommendations for RTH with a low to moderate load. However, since the used RTH design was neither effective nor superior, exercise variables and/or the hypoxic dose must be reconsidered.

### Effects of RTH on Muscle Strength Capacity

Regarding the muscle strength capacity (maximal isokinetic strength), we found only partially significant improvements (only for the muscles of the legs but not for the muscles of the arms with no differences between the groups RTH and RT within the age cohorts). The increase in the maximal isokinetic muscle strength of the legs was lower for both groups of the young people than in both groups of the older people. Considering the measurement error of the used device and method for maximal isokinetic strength testing (knee extension: 3%, knee flexion: 6%; see Törpel et al., 2017), these effects can be associated with the intervention RTH and RT. In contrast, the non-significant changes of the muscle strength of the arms were not higher than the measurement error (elbow flexion: ∼6%, elbow extension: ∼8%; see Törpel et al., 2017) and must therefore considered to be insubstantial. In comparison to previous RTH studies using low to moderate loads, the amount of these improvements are comparable (Manimmanakorn et al., 2013a, b; de Smet et al., 2017) or higher (Friedmann et al., 2003). Regarding muscle strength capacity, the RTH-interventions using a moderate to high load achieved comparable (Mayo et al., 2018), higher (e.g., Nishimura et al., 2010; Kon et al., 2014; Inness et al., 2016; Yan et al., 2016), and lower results (Ho et al., 2014a). Based on the higher number of moderate- to high-load RTH studies that improved muscle strength capacity, it can be supposed that the load is a crucial exercise variable for RTH for improving muscular strength in an effective way (both absolute and compared to resistance training under normoxia). The tendency to use higher loads in RTH also meets the recommendations for resistance training under normoxia (see Schoenfeld et al., 2016). However, with regard to the investigation by Scott et al. (2018), not only the load is important for RTH to be effective. Among other exercise variables, also (i) the inter-set pause (Scott et al., 2015a; Lockhart et al., 2018), (ii) type of resistance exercise (e.g., singe-joint vs. multi-joint resistance exercises; discussed by Lockhart et al., 2018, or whole-body vibration, Camacho-Cardenosa et al., 2019a), (iii) the length of the intervention period (known from resistance training under normoxia; Silva et al., 2014), and/or (iv) the movement velocity (known from resistance training under normoxia; Davies et al., 2017; Hackett et al., 2018) all seem to be crucial factors in improving the effectiveness of RTH. While in the current study the inter-set pause as well as the type of resistance exercise were designed in a way that a high efficiency could be expected, the intervention period could have lasted longer and the movement velocity could have been increased (i.e., instead to 2 s for the concentric and eccentric phase, just 1 s) to improve the effect of the used low- to moderate-load RTH design. However, further intervention studies are needed to verify this assumption.

### Effects of RTH on Cardiopulmonary Capacity as Well as the Systolic and Diastolic Blood Pressure

Even though the cardiopulmonary capacity of the group RTH was not superiorly improved compared to the group RT in both age cohorts (no significant time × group effects), the significant increase in the VO2_peak_ (+ 7.4%) and PO_max_ (+ 5.2%) as well as the significant decrease in the submaximal HR (at 1 W/kg, −1.9%) in the group RTH of the older cohort suggest that the RTH may have had a positive effect on the cardiopulmonary system. However, there was also an improvement of the VO2_peak_ of + 10.5% in the group RT of the older participants, which can be considered as practically relevant, although it was not statistically significant. In the young people, we observed a significantly improved exercise economy in the group RTH (VO2_1__W/kg_: −4.7%) and in the group RT (HR_1__W/kg_: −6.6%). Therefore, it appears that the resistance training itself also had a positive effect on the cardiopulmonary system of the young and older people.

The effectiveness of different types of resistance training (e.g., low load and high volume, high load and low volume) on the cardiopulmonary capacity (endurance performance) is positively associated with an increase of the VO2_peak_ and/or the PO_max_ between 1 and 8% in young people after several weeks of training (8 weeks to 6 months) (Bazyler et al., 2015). Comparable effects have also been proven for older people, however with no or only a moderate significance (Strasser et al., 2009). Therefore, with the comparatively short intervention period of 5 weeks in the present study, the shown effects reached a magnitude that was to be expected from the perspective of resistance training.

Additionally, previous studies that have investigated a low-load RTH (e.g., de Smet et al., 2017; 5 week intervention period, 3x/week, loads 20 to 25% of the 1RM) found no superior effect in comparison to a similarly designed resistance training under normoxia on the VO2_peak_ in young people. In contrast, the study by Álvarez-Herms et al. (2016) found a significant improvement in anaerobic performance (+ 53.8% for the total number of sets at 90% of the 300-m “all-out” test). This improvement was reached by performing a circuit resistance training with body weight exercises over 4 weeks (3x/week) under hypobaric hypoxia. The participants performed body weight resistance exercises with high speed/explosive movements leading to an increased ability of the type 2 muscle fibers to produce force (Claflin et al., 2011) and this is associated with better anaerobic performance (Kin-İsler et al., 2008; Alemdaroğlu, 2012). The authors discussed adaptations on the mitochondrial level (increased mitochondrial density), enzyme/metabolic level (increased monocarboxylate transporter 1 [MCT1], increased higher buffer capacity), and the cardiovascular level (increased stroke volume through a higher left-ventricular contractile force and/or through an increased cardiac filling pressure) (Álvarez-Herms et al., 2016). Hence, muscular and/or cardiopulmonary adaptations might be the reason for the improved anaerobic performance. Regarding the design of the resistance training, Álvarez-Herms et al. (2016) stated that the training was characterized by a “light load” because they used body weight resistance exercises. However, the amount of the relative load of body weight exercises depends on the maximum muscle strength capacity. Consequently, it might be that the load for the male 400-m elite athletes was higher (>50% of the 1RM, classification see Garber et al., 2011) which would mean that the type of this RTH-intervention must be defined differently (RTH using moderate to high loads). In addition, in comparison to the current study, the different type of resistance exercises as well as differently designed exercise variables (e.g., movement velocity, number of repetitions, inter-set pause) may have led to a different training stimulus. Due to those differences regarding the design of the resistance training as well as the different tests used (spiroergometry on a bicycle vs. 300-m “all-out” running test), a proper comparison between the present study and the study by Álvarez-Herms et al. (2016) is not possible.

RTH studies using moderate to high loads showed improvements in tests that are linked to the cardiopulmonary capacity (Álvarez-Herms et al., 2012: counter movement jumps for 60 s and determination of the heart rate recovery index; Ramos-Campo et al., 2018a: incremental treadmill test; Mayo et al., 2018: bronco test). In summary, it can be assumed that RTH has a positive effect on the cardiopulmonary system. However, it seems that it depends on the design of the resistance training (e.g., load, movement velocity) and on the length of the intervention period.

In addition to the effect on the cardiopulmonary capacity, the effect on the SBP and DBP in rest as well as during physical exercise were also investigated. Here, we observed a significant decrease of the SBP by 10 mmHg at rest and during physical exercise in the group RTH of the young participants. A reduction of the SBP could also be observed in the group RTH of the older people (rest −10 mmHg, 50 W −5 mmHg, 75 W ± 0 mmHg, 100 W −5 mmHg), but without a significant effect. No changes or slight decreases of maximal −5 mmHg were reached in the groups RT young and RT old. These changes of the SBP correspond to the extent that can be expected by resistance training (−4 mmHg; see Lemes et al., 2016). Due to the higher changes (significant and non-significant) of the SBP in both age cohorts that performed RTH, it appears that this type of intervention may have a higher vasodilation effect on the arteries than resistance training under normoxia. There is a good body of evidence which supports the statement that there is a generally higher effect on vascular health when exercise is performed under hypoxic conditions than under normoxic conditions (Montero and Lundby, 2016). Based on the knowledge that a decrease in the SBP by 2 mmHg would involve about 10% lower stroke mortality and about 7% lower mortality from ischemic heart disease or other vascular causes (Lewington et al., 2002), the reductions of the SBP achieved in the current study are crucial and practically relevant for health (independent if there are significant or non-significant). The increase in the DBP by + 2.5 mmHg during exercise in the group RT old is not practically relevant as the increase was no higher than 90 mmHg (Taylor et al., 2011; Currie et al., 2018).

### Effects of RTH on EPO, tHb, and Blood Volume

The increase in EPO after RTH in the young and older cohort is in line with previous investigations that researched the dose–response relationship regarding the duration of an hypoxic exposure and the EPO-expression (significant increase in EPO after a hypoxic exposure of 90 to 180 min; see Eckardt et al., 1989; Knaupp et al., 1992; Törpel et al., 2019). However, the acute increase in EPO was not largely different between the groups RTH and RT in both age cohorts. This can be explained by the wide natural range of the basal EPO level (6 to 32 U/l; Jelkmann, 1992) as well as the inter-individual increase of EPO in response to a hypoxic exposure (Jedlickova et al., 2003) which may have avoided the differences becoming statistically significant.

The intermittently higher EPO level, however, was not able to increase the erythropoiesis sufficiently for augmenting the tHb (and thus also the BV). The reduction in tHb in the RTH young (−2.5%) is not understandable for us because usually no change or a positive effect is associated with hypoxia (Płoszczyca et al., 2018) or exercise (Mairbäurl, 2013) regarding this parameter. It is known that the tHb increases by ∼1.1% per 100 h of hypoxic exposure (Gore et al., 2013). In addition to the total duration of a hypoxic exposure, the duration of the single hypoxic phases is also crucial to increase hematological parameters if intermittent hypoxic protocols are to be used. Here, a hypoxic exposure of at least 12 h per day is needed (Rusko et al., 2004; Wilber, 2007; Lobigs et al., 2018). Therefore, the total hypoxic duration of 60 h as well as the single hypoxic exposure of 3 h on 4 days per week were not sufficient to increase erythropoiesis. In RTH-studies there is only one study (de Smet et al., 2017) where such a long hypoxic exposure was used: 5 weeks, 5 days per week, ∼15.5 h per day hypoxic exposure. Here, a mean increase in tHb of 2.6% was observed (range 1.3 to 5.9%). Therefore, if clinicians or researchers aim to provoke hematological effects through RTH, the hypoxic dose should be designed as described. Due to the much higher hypoxic exposure needed to achieve hematological effects, we would recommend limiting the hypoxic exposure to the duration of the resistance training (including a 10-min acclimatization to the hypoxia, warm-up, and cool-down) in future RTH-intervention studies.

### Effects of RTH on Body Composition

Body composition, and in particular the FFM or lean body mass, was analyzed in previous RTH studies to indirectly research the effect of this type of intervention on muscle mass/muscular hypertrophy (Ho et al., 2014b; Kon et al., 2014; Chycki et al., 2016; Yan et al., 2016; Martínez-Guardado et al., 2019). We did not observe any changes regarding the FFM which is in line with the insights from the meta-analysis by Ramos-Campo et al. (2018b) stating that there is only a small to non-significant effect regarding the increase of the muscle size with RTH compared to RT under normoxia. However, the general time course of muscle hypertrophy by RT is not exactly known as it is a function of different factors [e.g., design of the RT (Wernbom et al., 2007), age (Degens and Alway, 2003), nutrition (Stokes et al., 2018)]. After a resistance training of few weeks (4 to 8 weeks), like in RTH studies, only a small increase in muscle size is to be expected (Damas et al., 2018). Therefore, RTH studies should be performed over a longer intervention period (+ 12 weeks) to investigate the effect on muscle hypertrophy more adequately. Even though the exposure to hypoxia as well as exercise under hypoxia is positively associated with the loss of body fat (Netzer et al., 2008, 2015), no changes of the FM were achieved in the current study. This result is in line with previous RTH studies that also did not find any effects on body fat (Ho et al., 2014b; Chycki et al., 2016; Martínez-Guardado et al., 2019). There are two RTH studies by Kon et al. (2014) and Yan et al. (2016) showing a significantly reduced FM between −1 and −2%, but without significant differences to the group who performed resistance training under normoxia. Based on those results, it seems that RTH after an intervention period of 5 to 8 weeks has no or no additional effect on the reduction of body fat.

### Hypoxic Dose of RTH

The internal hypoxic intensity (= SpO_2_) during the IHE and RTH phase met the predefined hypoxic intensity (see Figure 4 and chapter *Materials and Methods*). Even though the differences in the SpO_2_ were only small (SpO_2_ 2 to 3%), the SpO_2_ was still significantly lower in the young than in the older age cohort during the IHE and the RTH phase in the second to the fifth week. The hypoxic intensity is crucial regarding the strength performance (mean force, peak force) and might therefore also be crucial for adaptational processes. While Ramos-Campo et al. (2017) showed an enhanced performance response (meaning a lower mean force and peak force), Scott et al. (2015b) observed an unaltered performance response under different external hypoxic levels (FiO_2_ 13 vs. 16%). However, both studies used different exercise variables. Thus, it can only be assumed that the hypoxic intensity can be decisive regarding strength performance, but does not have to be (depending on the design of the resistance training). In addition, Scott et al. (2018) did not find any differences in muscle activation and blood lactate concentration during and after a RTH with high loads under moderate-level hypoxia (FiO_2_ 16%, SpO_2_ ∼86%) vs. high-level hypoxia (FiO_2_ 13%, SpO_2_ ∼73%) (and also vs. normoxia). Here, the difference between the internal hypoxic intensity was much higher than in our study. Although we cannot be certain, based on the small (albeit significant) differences in the internal hypoxic intensity in the current study, we are convinced that these differences were not practically crucial, because the stimuli for adaptation processes were different between young and older people.

### Limitations

We recruited both females and males for the young and older age cohort. While the sample size of females and males was largely balanced in both age cohorts, the number of males in the young cohort was higher than the number of females leading to a heterogenous distribution of sex across the four groups (RTH-young, RT-young, RTH-old, RT-old). Given that gender can influence adaptational processes (Folland and Williams, 2007; McMahon et al., 2018) and thus also study results, data analysis between age-cohorts should be handled with care in this study. Future studies should take into account that each gender should be investigated separately, cohorts should be balanced regarding the sample size of females and males, or the total sample sizes of experimental groups should be sufficiently large.

## Conclusion

RTH used with low to moderate loads had no additional effects on the muscular strength capacity, cardiopulmonary capacity, EPO, tHb, blood volume, fat free mass, or fat mass in young people and older people. However, this statement can only be applied for RTH designed as in this study and after an intervention period of 5 weeks. Based on previous RTH-studies as well as on the knowledge from resistance training studies in general, it can be assumed that the expected higher effects can still be achieved with the change of exercise variables of RTH (e.g., longer intervention period, higher movement velocity, higher loads). Therefore, further RTH intervention studies (particularly those with longer intervention periods) are strongly recommended to reinforce knowledge about the adaptational processes and the effects of this type of resistance training in young and older people.

## Data Availability Statement

The datasets generated for this study are available on request to the corresponding author.

## Ethics Statement

The studies involving human participants were reviewed and approved by Ethical Committee of the Otto von Guericke University Magdeburg/Germany (Nr. 74/15). The patients/participants provided their written informed consent to participate in this study.

## Author Contributions

AT and LS contributed to the study design. AT contributed to the recruitment of participants, implementation of the intervention, data analysis, and manuscript drafting. AT and BP contributed to the diagnostic. LS and BP contributed to the review of the drafted manuscript.

## Conflict of Interest

The authors declare that the research was conducted in the absence of any commercial or financial relationships that could be construed as a potential conflict of interest.
